# Solution Synthesis of NdTe_3_ Magnetic Nanosheets

**DOI:** 10.1021/acs.chemmater.4c01362

**Published:** 2024-07-04

**Authors:** Joel Swanson, Salah Eddin El Jamal, Tyler Hartman, Orlando C. Stewart, Priscilla Glaser, Adam J. Biacchi, DaVonne Henry, Amy Liu, Sarah L. Stoll

**Affiliations:** † Department of Chemistry, Georgetown University, 37th and O Sts. NW, Washington, D.C. 20057, United States; ‡ Nanoscale Device Characterization Division, National Institute of Standards and Technology (NIST), 100 Bureau Dr., Gaithersburg, Maryland 20899, United States; § Department of Physics, Georgetown University, 37th and O Sts. NW, Washington, D.C. 20057, United States

## Abstract

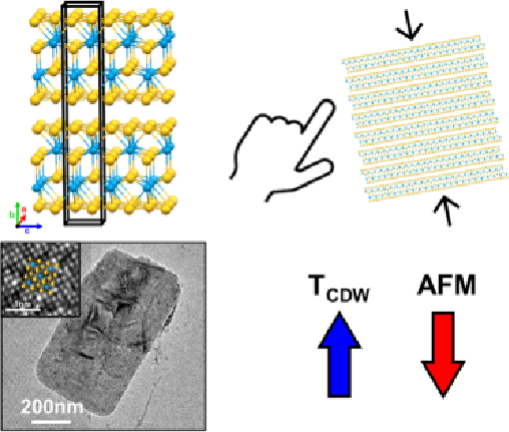

Neodymium tritelluride is a layered van der Waals material, with correlated electronic properties including high electronic mobility, charge density waves, and antiferromagnetism. We developed a solution synthesis method to form free-standing nanosheets of NdTe_3_, with nanosheet lateral dimensions of 200–400 nm. The morphology of the nanosheet was influenced by the neodymium precursor. When Nd[(N(SiMe_3_)_2_]_3_ was used as the metal source the nanosheet thickness average was 12 ± 2.5 nm, alternatively the combination of NdCl_3_ and Li(N(SiMe_3_)_2_) led to thicker nanosheets, approximately 19 ± 2.4 nm. We believe that the difference in thickness and changes in surface chemistry point to the role of chloride in accelerating nanocrystal growth for the synthesis with NdCl_3_ (and Li(N(SiMe_3_)_2_). Both types of nanosheets exhibit charge density wave (CDW) distortions as measured using electron diffraction and investigated using variable temperature Raman scattering. Interestingly, the magnetic studies suggest a distinct change in properties between 12 and 19 nm thickness in antiferromagnetic NdTe_3_.

## Introduction

Inspired by graphene, the most recent class of atomic crystals to be investigated in two-dimensions are the magnetic van der Waals materials.^1^ The evolution of magnetic properties in 2D materials as a function of thickness is thought to depend on the spin-dimensionality and magnetic anisotropy of the material.^2^ In theory, low dimensional magnets are predicted to have significantly reduced magnetic ordering temperatures and increased anisotropy.^3^ In practice, changes to the ordering temperature and magnetic orientation as a function of thickness is very material-dependent. Recent studies have discovered intrinsic ferromagnetism,^4^ layer-dependent magnetism,^5^ thickness- and field-dependent ordering temperatures (*T*_c_),^6^ and also room temperature ferromagnetism in the monolayer.^7^ The control over properties in two-dimensional magnets such as gate-tunable room-temperature ferromagnetism,^6^ large tunneling magnetoresistance,^8^ spin-filtering effects,^9^ and topological spin textures such as skyrmions^10^ suggest novel device applications.^1^

One limitation to advancing understanding of 2D magnetism is the synthesis of ultrathin materials with controlled dimensions, high crystallinity, and yields. Studies of high-quality monolayer materials requires expensive and time-intensive synthetic techniques such as molecular beam epitaxy (MBE). In addition, single layer nanosheets demand new characterization methods to determine the magnetism due to the low volume of material.^11^ Gas-phase deposition of thin films have similar disadvantages to MBE, including low yields, high temperatures, and the influence of the substrate on properties. Free-standing nanosheets by mechanical exfoliation is an alternative synthetic route ideal for high-performance devices,^12^ but exfoliated flakes have highly variable thickness, ranging from single layer to 100 nm nanosheets.^13^ By contrast, liquid exfoliation methods produce suitable yields for magnetic characterization, but often require intercalation to weaken the interlayer adhesion,^14^ leading to structural instabilities.^15^ Constrained by synthetic requirements, the focus has been on intrinsically magnetic van der Waals materials and dominated by transition metal compounds.^16^ However, underexplored lanthanide materials have advantages due to the large saturation magnetization values, enhanced magneto-crystalline anisotropy, and potential magneto-optical or magneto-resistive effects.^17^ There are a limited number of examples to generalize, but the metalloxenes, MSi_2_ and MGe_2_, for M = Eu and Gd, exhibit an interesting shift from 3D antiferromagnetism in the bulk to 2D ferromagnetism in nanosheets.^18^ Despite these advantages and promising properties, relatively few lanthanide compounds have been studied.

**Figure 1:**
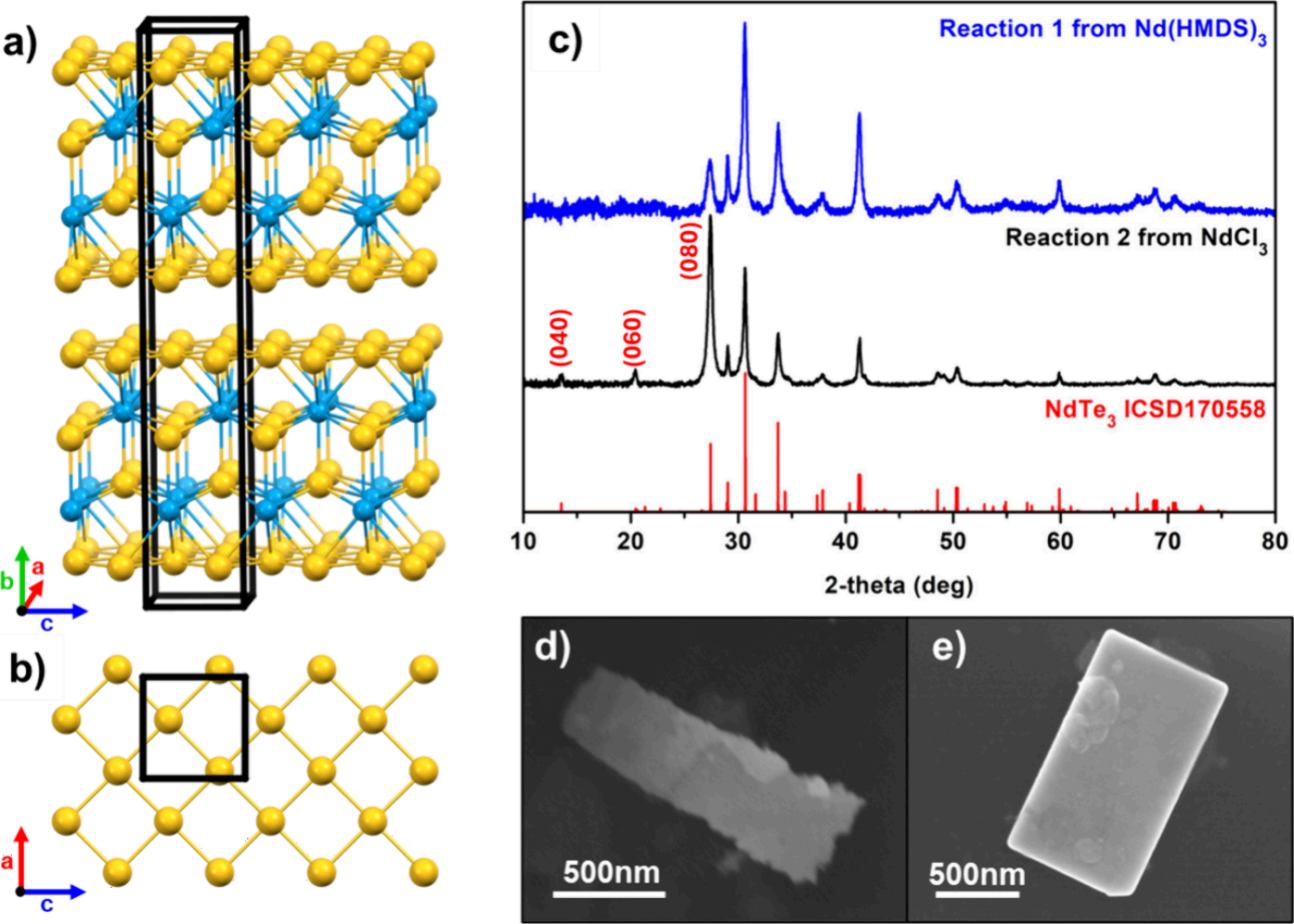
Lanthanide tritelluride crystal structure showing the layer stacking (a) and a top-down view of the telluride layer (b). Powder X-ray diffraction patterns of each reaction with NdTe_3_ reference (c); scanning electron microscopy images of NdTe_3_ nanosheets from reaction 1 using Nd(HMDS)_3_ (d) and reaction 2 using NdCl_3_ (e).

The lanthanide tritellurides, LnTe_3_, form an important class of rare-earth magnetic van der Waals materials. The bulk materials exhibit correlated electronic properties including charge density waves, high electron mobility, and a competition between superconductivity and antiferromagnetic ordering.^19,20^ The structure, as shown in Figure 1, is composed of magnetic, insulating [LnTe]^+^ layers, separated by two metallic sheets of [Te_*n*_]^−0.5^. The differences in bonding leads to highly anisotropic electron conductivity, with an increase in resistance approximately 2 orders of magnitude perpendicular to the Te layers.^21,22^ The telluride layers also host charge density waves (CDWs), which are formed through the condensation of the pseudo-square lattice into telluride oligomers.^23^ The CDW is stable to unusually high temperatures, ranging from >500 K for LaTe_3_^24^ down to 244 K for TmTe_3_,^25^ where the transition temperature varies regularly with lanthanide ionic radii. The structural and electronic anisotropy is echoed in the magnetic properties, where the magnetic moment aligns in the [LnTe]^+^ plane for Ln = Ce, Nd, and Sm.^26,27^ Of these materials, neodymium has the highest ordering temperature and most stable trivalent oxidation state.

Prior studies of ultrathin nanosheets of the lanthanide tritellurides are limited to GdTe_3_ and TbTe_3_, prepared by mechanical exfoliation. This method is ideal for conductivity studies; the GdTe_3_ nanosheets (22 nm thick) were found to maintain the high carrier mobility of the bulk, competitive with black phosphorus,^25^ while TbTe_3_ nanosheets (23 nm thick) were found to have extremely large magnetoresistance (as high as 5600%).^28^ Solution methods, such as liquid exfoliation, can produce nanosheets in this range and has been reported for lanthanide telluride nanosheets for the nonmagnetic LaTe_3_ and HoTe_3_.^29^ However, liquid exfoliation led to poor crystallinity, agglomeration, and a broad size distribution.^30^ Colloidal routes to nanosheets can produce high yields but importantly have demonstrated potential to form highly uniform nanosheets with controlled thickness,^31,32^ down to the monolayer.^33^

Colloidal synthesis provides important complementary information to studies of monolayer two-dimensional materials as well as a route to more scalable materials.^34^ Colloidal synthesis of two-dimensional nanosheets is not limited by the type of material, so this method should expand the classes of magnetic materials for 2D magnetic studies.^35^ Although colloidal syntheses produce surfactant-coated nanosheets, which can inhibit ohmic contact, it is possible to produce smooth films by drop casting and annealing for electronic measurements.^36^ Colloidal materials are appropriate for optical methods (including magnetic circular dichroism (MCD), magneto-optical Kerr effect, and Raman spectroscopy) that provide indirect information about the magnetism of two-dimensional materials.^1^ The increased yields from solution syntheses have the advantage of direct magnetic characterization using magnetic susceptibility. To our knowledge the colloidal synthesis of lanthanide tritellurides has not been reported, which we attribute to a lack of suitable precursors.

We discovered a solution route to form LnTe_3_ nanosheets and reported the synthesis of highly crystalline NdTe_3_ ultrathin nanosheets. Using two closely related synthetic methods, the nanosheets had similar lateral dimensions (200–400 nm), but measurably different nanosheet thicknesses. The first approach used a neodymium precursor, Nd[(N(SiMe_3_)_2_]_3_, forming nanosheets averaging ∼12 ± 2.5 nm thick, whereas the *in situ* reaction of NdCl_3_ and Li(N(SiMe_3_)_2_ led to nanosheets with an average thickness of ∼19 ± 2.4 nm, with very different morphologies. The nanosheet thicknesses were determined by powder diffraction and compared to measurements by transmission electron microscopy (TEM) and atomic force microscopy (AFM). To gain insight into the mechanism of nanosheet growth, the surface and composition were determined by a combination of energy-dispersive X-ray spectroscopy (EDS) and X-ray photoelectron spectroscopy (XPS). Both nanosheets exhibited CDWs based on electron diffraction and temperature-dependent Raman studies. Importantly, we found that the two nanosheets exhibited different magnetic properties, based on magnetic susceptibility.

## Results and Discussion

### Nanosheet Synthesis

The successful synthesis of NdTe_3_ from solution was exciting in light of the difficulty in forming lanthanide-tellurium bonds, disfavored due to the hard–soft mismatch between the two elements. We have previously found that lanthanide chalcogenide single-source precursors circumvent this problem,^37^ but unfortunately tellurium ligands are challenging to synthesize, and the yields of the few known complexes are typically low.^38^ Thus, we have adopted a solution synthesis using separate lanthanide and telluride sources. Our criteria for the lanthanide precursor were high solubility and reagents that exclude oxygen containing anions (e.g., nitrate or triflate) or ligands (carboxylates, acetylacetonate), to avoid oxide impurities. We chose neodymium tris(hexamethyldisilylamide), Nd((Me_3_Si)_2_N)_3_ (denoted here as Nd(HMDS)_3_), which is soluble in nanoparticle solvents and commercially available. The silylamide has been used in the synthesis of metal nanoparticles for similar reasons, high solubility, and limited potential for oxidation.^39^ The success of metal silylamide reagents has led to different rationales for what has become known as “amide assisted” nanoparticle syntheses.^40^ To avoid the need for synthesizing Nd(HMDS)_3_, we also considered what appeared to be an equivalent approach via the *in situ* reaction between NdCl_3_ and Li(HMDS). Transition-metal–telluride nanoparticles are sensitive to the metal precursor leading to different phases,^41^ but in this case, there was no difference in the product phase, NdTe_3_. However, there were distinct differences in the nanosheet thickness, morphology, and surface chemistry as described below.

There has been a notable effort to identify tellurium reagents for colloidal nanoparticle synthesis, but because of the low electronegativity of tellurium and high stability of elemental Te^0^, alternative methods have been explored such as electrodeposition, microwave, or solvothermal synthesis.^42^ Trioctylphosphine telluride (TOP-Te) is a tellurium source that can act as a neutral tellurium transfer reagent,^43^ but we find that its reaction with Nd(HMDS)_3_ only leads to an amorphous product or Te metal. Building on work by Krauss, who found enhanced reactivity of TOP-Se when paired with secondary phosphines,^44^ we found that the combination of trioctylphosphine telluride (TOP-Te) with diphenylphosphine (HPPh_2_) and Nd(HMDS)_3_ in oleylamine cleanly forms NdTe_3_. Using the nonmagnetic La(HMDS)_3_ as a model, we identified trace amounts of [Te_2_PPh_2_]^−^ and [TePPh_2_]^−^ anions based on ^31^P NMR studies (SI-1),^45^ which may be the reactive tellurium species in solution.^46^ It is possible to trace the color of this reaction from the initial red/black precursor solution to the characteristic gold color of the LnTe_3_ family.

### Phase Determination

The NdTe_3_ nanosheet powders exhibit phase-pure powder X-ray diffraction (PXRD) patterns that index to ICSD-170558, as shown in Figure 1, based on peak position and relative intensity. The preferred orientation is notable for the sample from NdCl_3_ (the second synthesis) in the enhanced peaks along the unique axis (080), (060), and (040) peaks. The most common impurity we have observed in lanthanide telluride nanoparticle synthesis is elemental Te, which inconveniently has its most intense peak overlapping with with the 080 peak for NdTe_3_. However, the other characteristic peaks for this phase are distinguishable and absent in both syntheses (NdTe_3_ from Nd(HMDS)_3_ and NdCl_3_). In addition, tellurium metal was not observed in Raman studies, and elemental maps of the nanosheets suggest that tellurium is always colocated with neodymium. No other crystalline phases are observed based on powder diffraction.

### Nanosheet Dimensions

**Figure 2:**
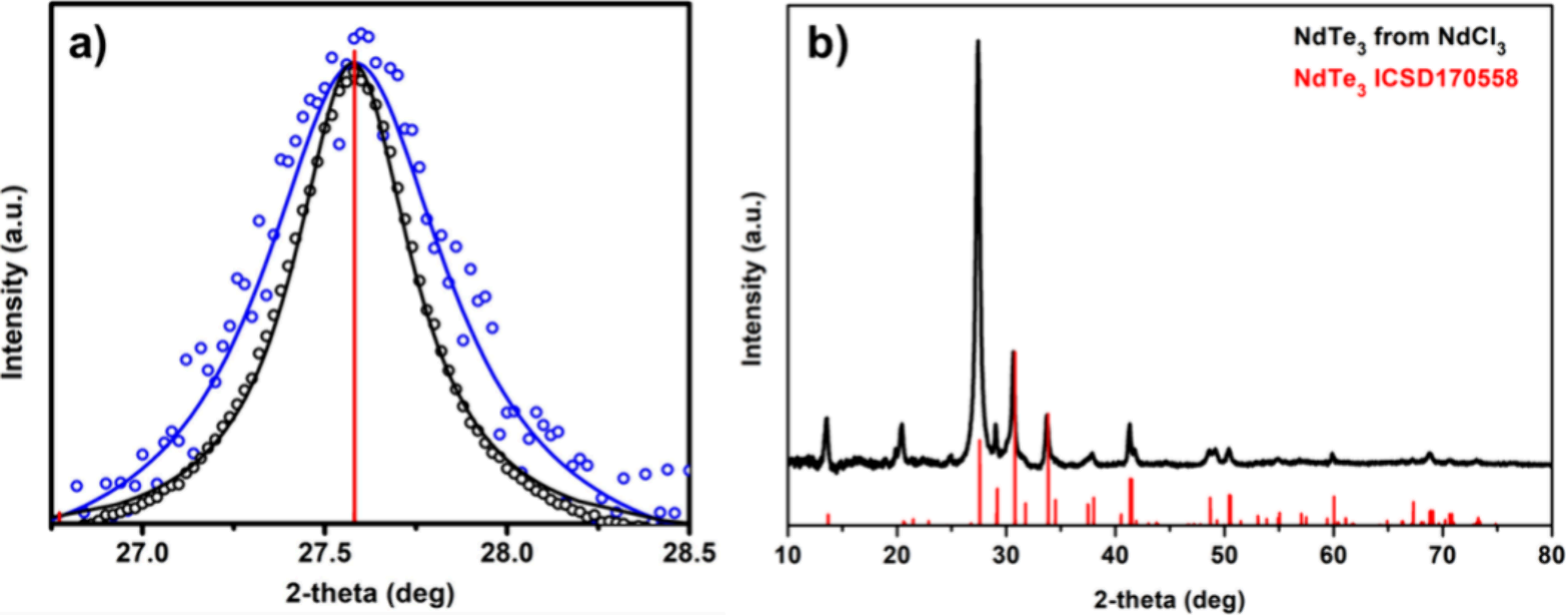
Powder X-ray diffraction of NdTe_3_ nanosheets, (a) thick (black) and thin (blue) compared to the reference line along the NdTe_3_ {080}. The experimental data is in empty circles and the associated Lorentzian fit is in the solid lines. (b) Preferred orientation of thick NdTe_3_.

To determine the average thickness of the nanosheets, we used the Scherrer equation for the line broadened reflection along the unique axis of the material (the *b* axis) in the PXRD as shown in Figure 2. Drop cast films of the NdTe_3_ sample made from Nd(HMDS)_3_, showed modest preferred orientation in the PXRD, and using the line broadened (080) of 8 samples, led to a calculated average thickness of 12 ± 2.5 nm. This corresponds to approximately 4 unit-cell thick nanosheets. Comparatively, drop cast films of the NdTe_3_ nanosheets synthesized from NdCl_3_ showed much greater preferred orientation along the (0*k*0) direction in PXRD (enhancing the 080, and more visible 040 and 060 peaks) but much less line broadening. Based on the Scherrer equation for the (080) reflection of 6 samples, the average thickness was calculated to be measurably larger at 19 ± 2.4 nm, corresponding to ∼7 unit cells. We use this value as the most statistically relevant and conservative estimate of the average nanosheet thickness and conclude that the thickness of the NdTe_3_ samples made from Nd(HMDS)_3_ were distinct and thinner than NdTe_3_ from NdCl_3._

**Figure 3:**
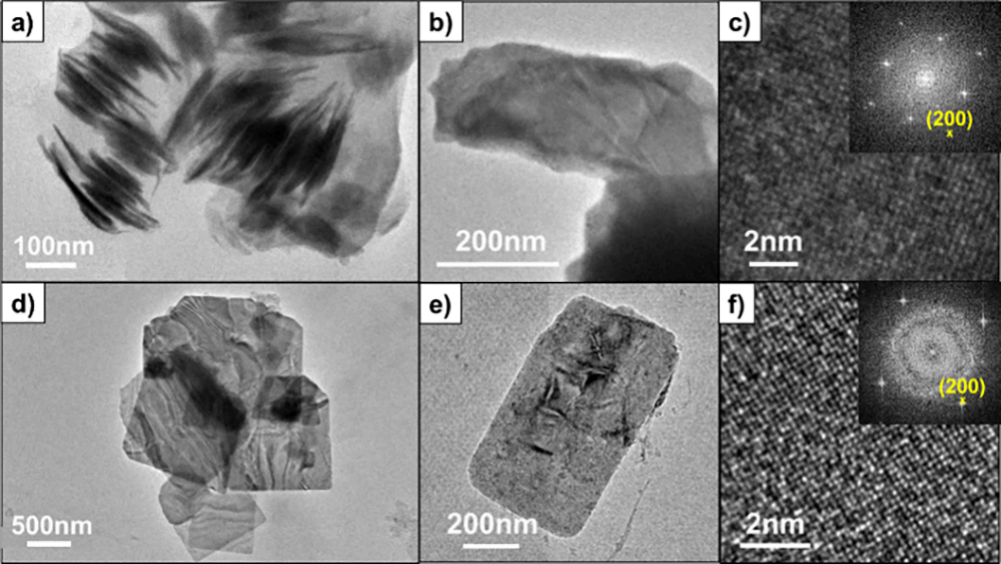
Low-magnification TEM images of thin NdTe_3_ nanosheets from Nd(HMDS)_3_, (a,b) and thick NdTe_3_ nanosheets from NdCl_3_ (d,e). High-resolution and (inset) fast Fourier transforms are shown for thin NdTe_3_ nanosheets (c) and thick NdTe_3_ nanosheets (f).

Both transmission electron microscopy (TEM) and atomic force microscopy (AFM) were also used to investigate the nanosheet dimensions, and the results are consistent with the PXRD results as summarized in Table 1. The thin NdTe_3_ samples made from Nd(HMDS)_3_ had distinct morphology by TEM with regions of wispy stacks, and the appearance of wet tissue (Figure 3 and SI-4). Single crystals of the lanthanide tritelluride materials are soft, due to the interlayer VdW bonding. While the TEM of the thicker sample exhibited a few wrinkles, the thinner material appeared to be highly buckled and folded, which may explain the reduced preferred orientation of the thinner material. From the TEM of a region of stacked nanosheets, it was possible to estimate the thickness of the NdTe_3_ from Nd(HMDS)_3_ as approximately 11 ± 4 nm, which is consistent with the PXRD (12 nm). The thicker NdTe_3_ (from NdCl_3_) exhibited preferred orientation in both the PXRD and TEM, which made it difficult to find by microscopy a nanocrystal oriented with a measurable edge to determine thickness (Figure 3, SI-3). The lateral (face) dimensions based on TEM were not notably different between the two syntheses of NdTe_3_ but were polydisperse and averaged to >400 nm based on measurements of ∼50 nanosheets.

**Table 1:** Summary of Nanosheet Dimensions

	**NdTe**_**3**_**from Nd(HMDS)**_**3**_		**NdTe**_**3**_**from NdCl_3_and Li(HMDS)**	
measurement method	length (nm)	thickness (nm)	length (nm)	thickness (nm)
Scherrer size		12 ± 4.1		19 ± 2.4 (080)
TEM	379 ± 319	11.3 ± 4.1	416 ± 155	
AFM (statistical)	282 ± 252	7.9 ± 5.5	295 ± 149	13 ± 8.3
AFM (gauss)	164 ± 74	5.3 ± 2.5	254 ± 134	11.5 ± 4.7

**Figure 4:**
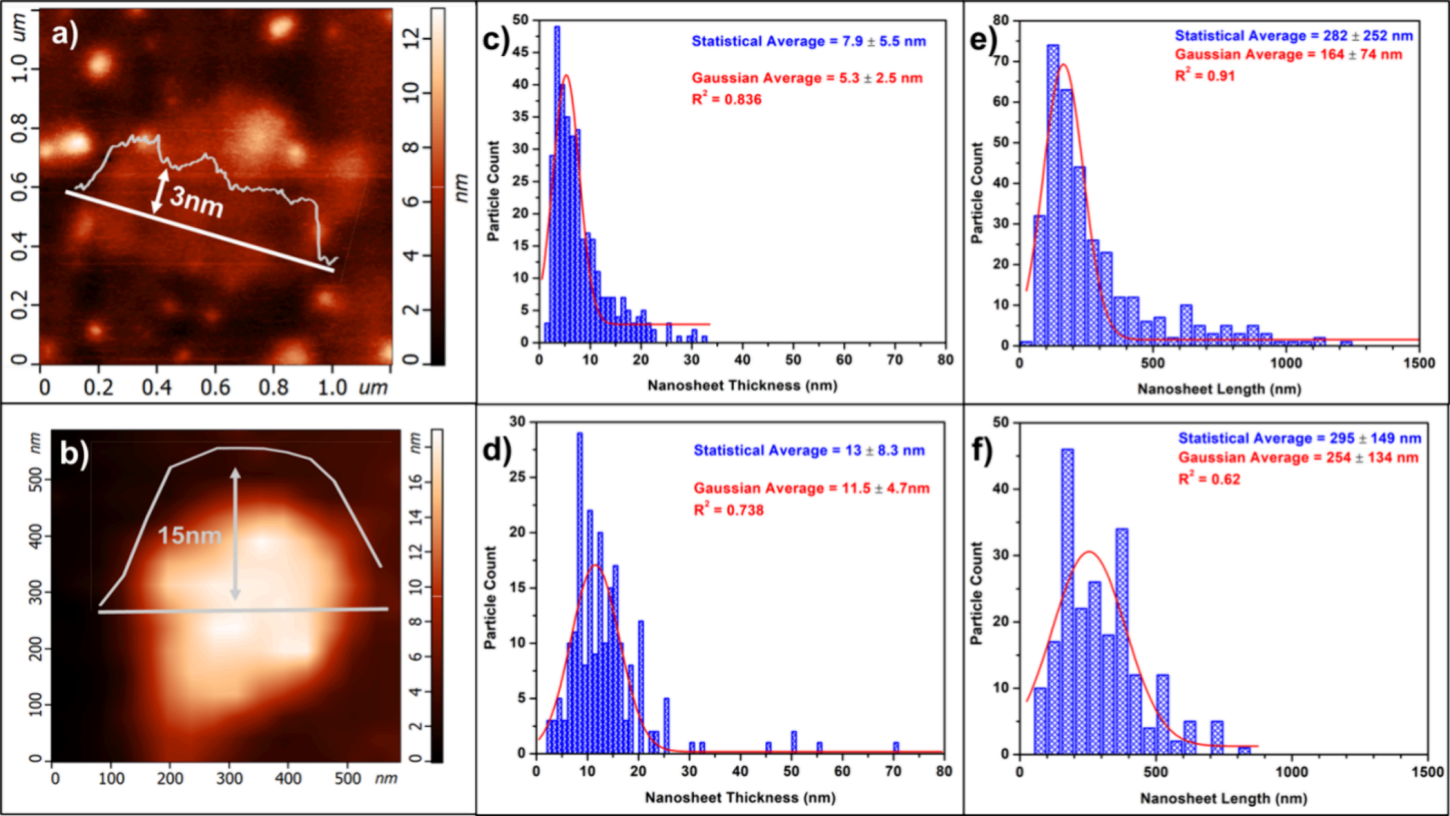
AFM images and histograms of thin NdTe_3_ nanosheets from Nd(HMDS)_3_ using Nd(HMDS)_3_ (a, c, e) and thick NdTe_3_ nanosheets from NdCl_3_ (b, d, f).

Although the sample size is much smaller for AFM, we attempted to use >200 nanocrystals for analysis of the dimensions, as shown in Figure 4. The AFM analysis suggest lateral dimensions were on average smaller and polydispersed (250–400 nm) for both nanosheets. The AFM thicknesses followed the same trend as the PXRD of the two samples. AFM found the NdTe_3_ from Nd(HMDS)_3_, the thinner sample to have a thickness of ∼8 ± 5.5 nm, in the same range as determined by PXRD (12 ± 2.5 nm). AFM analysis of the NdTe_3_ from NdCl_3_ was determined to have a thickness of 13 ± 8 nm, which is larger than the NdTe_3_ from Nd(HMDS)_3_ but statistically similar to that determined by PXRD (19 ± 2 nm).

We observed two different thicknesses of NdTe_3_ nanosheets from our two synthetic methods. While there is some variation in thickness comparing across methods, the trend when comparing the same method for each of the two syntheses is consistent; the NdTe_3_ nanosheets from Nd(HMDS)_3_ were thinner than the NdTe_3_ from NdCl_3_. We denote these two samples as thin and thick NdTe_3_ nanosheets, respectively, and use the 12 and 19 nm thickness from PXRD as the most representative measurement.

### Composition and Surface Characterization

**Figure 5:**
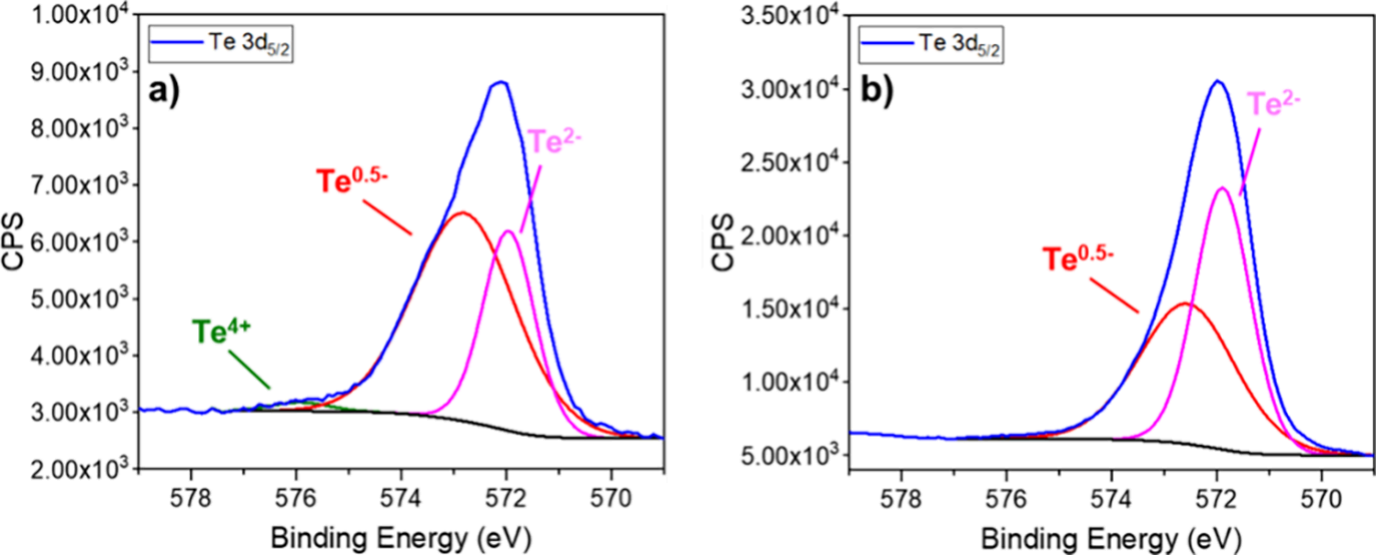
(a) Te 3d XPS for NdTe_3_ from Nd(HMDS)_3_ (b) and Te 3d XPS for NdTe_3_ from NdCl_3_. XPS samples were ion-sputtered prior to measurement.

To determine the composition of the nanosheets, we used energy-dispersive X-ray spectroscopy (EDS), which allows for elemental mapping. In addition, we also used X-ray photoelectron spectroscopy (XPS), which has the advantage that the peaks are sensitive to bonding and oxidation state. Based on EDS and XPS analysis of the sputtered NdTe_3_ nanosheets, there was no evidence of Li from the Li(HMDS) used in the synthesis of the thicker NdTe_3_ nanosheets. The lanthanide tritellurides exhibit air sensitivity, and the first evidence of oxidation is TeO_2_, which may be observed in the Raman spectroscopy^25,47^ and here evidenced in the XPS as Te(IV) (SI-2). It was possible to remove this surface oxide by sputtering with argon ions, simplifying spectra for analysis of the tellurium oxidation state (Figure 5). Although compositional analysis is approximate in the EDS, the NdTe_3_ from Nd(HMDS)_3_ was slightly above stoichiometric (Nd:Te was 1:3.4). The XPS exhibited weak evidence of Te(IV), potentially due to a small amount of surface oxidation. The EDS of NdTe_3_ nanosheets from NdCl_3_ was comparatively tellurium deficient (Nd:Te was closer to 1:1.5). Another notable difference in the EDS elemental mapping was the presence of chloride, which uniformly coats the faces or lateral surface of the thick nanosheets from NdCl_3_ (see Figure 7).

In addition, the XPS confirmed differences in the tellurium region for both the thin (from Nd(HMDS)_3_) and thick (from NdCl_3_) NdTe_3_. The crystal structure of NdTe_3_ has alternating layers [NdTe]^+^ and double [Te_*n*_]^−0.5^ layers as shown in Figure 1. Thus, there are two types of tellurium: Te^2–^ in the [NdTe] layer (ionic bonding) and Te^–0.5^ within the [Te_*n*_] double layer (“metal-covalent” bonding).^48^ The sputtered XPS data in Figure 5 indicate two types of tellurium, and the thin nanosheets (from Nd(HMDS)_3_) had a ratio of Te^2–^:Te^0.5–^ that was approximately 1:2, consistent with that expected for NdTe_3_. This supports the conclusion that the extent of surface oxidation is small, otherwise the ratio would be different. However, the thicker sample (from NdCl_3_) had a ratio closer to 1:1, which we attribute to a reduced amount of Te^–0.5^ based on the lower Nd:Te ratio from EDS.

**Figure 6:**
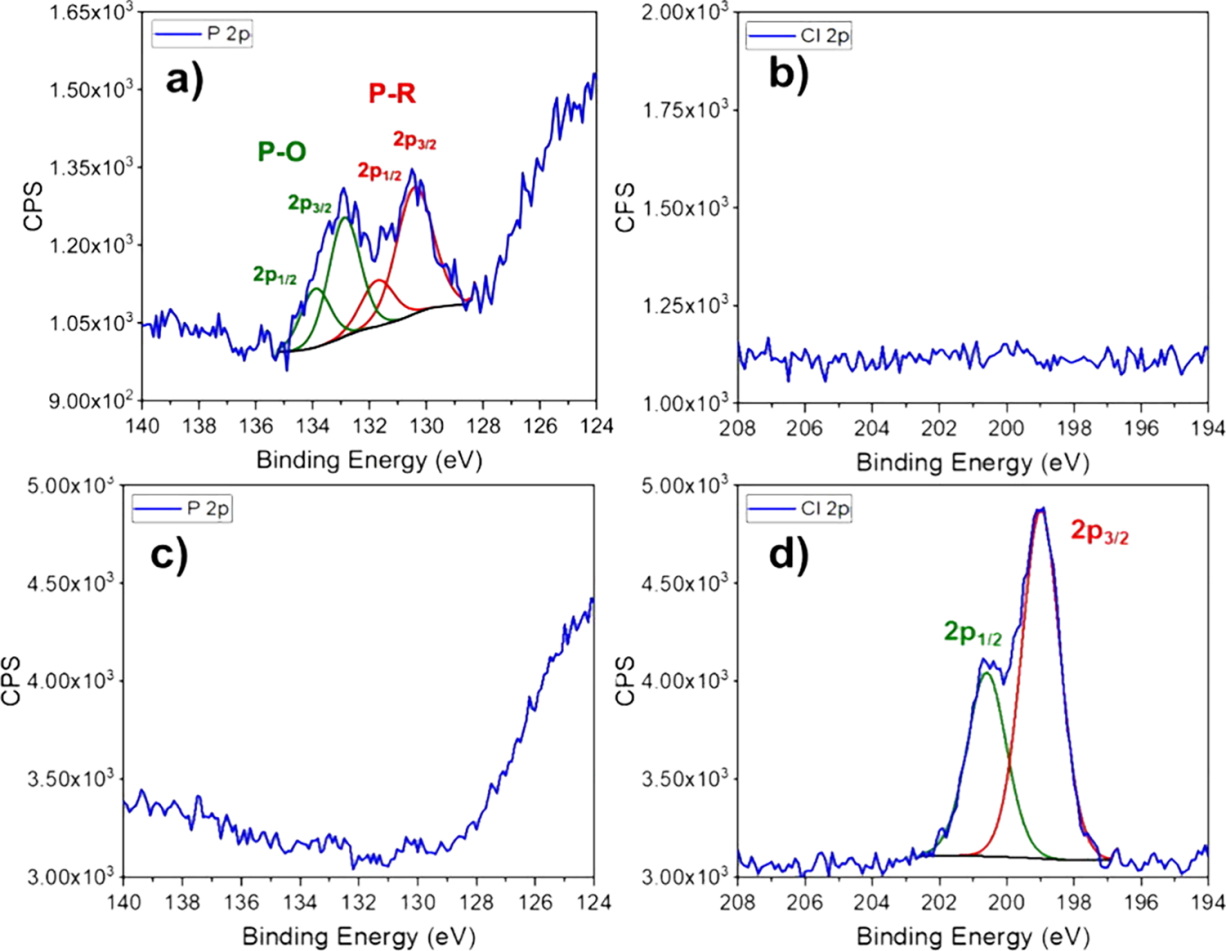
XPS studies of NdTe_3_ nanosheets. NdTe_3_ synthesized from reaction 1 using Nd(HMDS)_3_ (a,b) and from reaction 2 using NdCl_3_ + Li(HMDS (c,d). Samples were ion sputtered prior to measurement.

**Figure 7:**
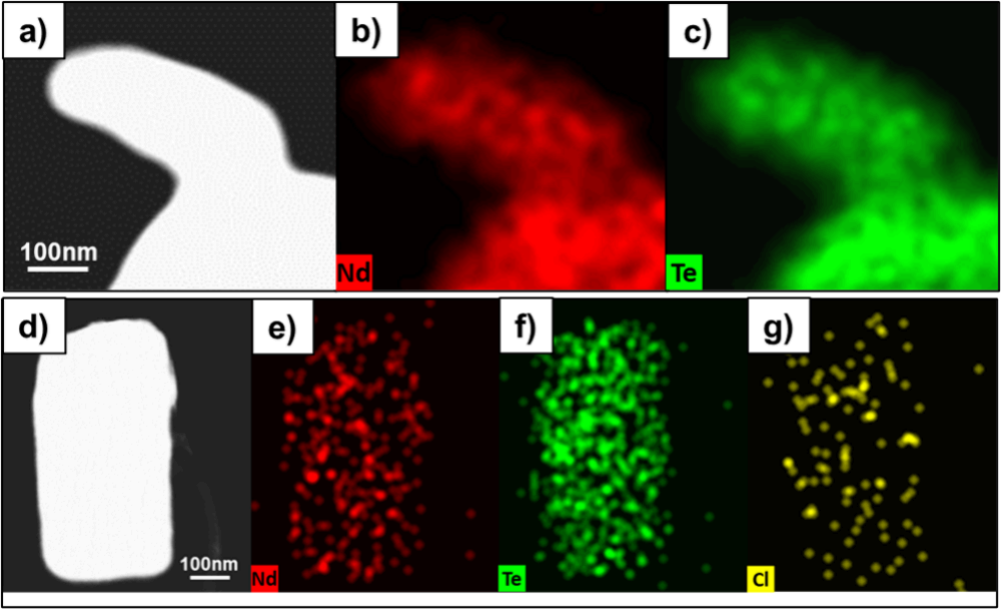
EDS maps of NdTe_3_ nanosheets from reaction 1 using Nd(HMDS)3 (a–c) and reaction 2 using NdCl_3_ + Li(HMDS) (d–g).

There is no evidence of impurity phases in the diffraction pattern, TEM, or Raman studies, so we infer that additional elements observed in EDS or XPS reflect the nanosheet surface. The solubility of both products in organic solvents indicate oleylamine as the dominant capping ligand, and supported by the FTIR spectra. Both EDS and XPS of the thicker sample (from NdCl_3_) found chloride across the surface (Figures 6d and 7g). The presence of Cl from the NdCl_3_ at the surface of the thick nanosheets is not surprising, but we suggest is consistent with a model in which these nanosheets are terminated by an ionic layer [Nd^3+^Te^2–^]^+^[Cl]^−^ rather than the expected van der Waals [Te_*n*_]^−^ layer. As a terminating ionic layer, the face of the distorted Nd–Te rock salt structure would have sites for direct coordination of Cl^–^ (and oleylamine) to Nd, whereas Cl^–^ is unlikely to bond to the [Te_*n*_]^−^ layer. This model also agrees with the lower Te^–0.5^:Te^2–^ ratio, which was closer to 1:1 by XPS. What was surprising is the XPS of the thin NdTe_3_ (from Nd(HMDS)_3_) had evidence of phosphorus (P), while the thick NdTe_3_ (from NdCl_3_) did not, see Figure 6a,c. We attribute to the P to trioctylphosphine as a capping ligand, but both syntheses used trioctylphosphine. All of the ligands, TOP, oleylamine, and chloride can bond to Nd, but the expected pattern is that the soft TOP should be the weakest for the hard Nd and easily outcompeted by Cl^–^ and oleylamine. Conversely, the soft Te is more likely to bond to TOP, suggesting that the thin material terminates in the expected [Te_*n*_]^−^ layer. There is an expectation that due to the weaker bonding across the van der Waals gap, that like exfoliated nanosheets, the solution grown nanosheets will have tellurium as the terminating layer (the lateral face of the nanosheet formed of the Te net). An interesting consequence of solution grown nanosheets is that there is no requirement that the surface layer to be capped on each face by one side of the van der Waals layer, unlike exfoliation methods. This may present opportunities to use surface modification to enhance the nanosheet magnetic properties.^49^

### Mechanism of Nanosheet Growth

Most solution grown nanosheets target layered materials, with the assumption that the directional crystal growth necessary to form nanosheets is facilitated by strong differences in bonding, strong within the layer but weak between layers. However, even highly anisotropic materials, such as the van der Waals transition-metal dichalcogenides, with controlled solution conditions, still often form multilayered nanomaterials.^50^ Identifying the solution conditions to control the growth of specific crystallographic faces is an important challenge in nanomaterials synthesis. One approach is to slow the overall rate of nanocrystal growth, which can enhance natural differences in crystal face energies and lead to anisotropic growth. This can be accomplished either through low temperatures, the slow injection of precursors, slow chalcogen generation, or highly coordinating solvents to slow nanocrystal growth.^51,52^ Alternatively, identifying a capping ligand with different facet binding energies can also enhance selective anisotropic growth.^53^

The silylamide anion was present in both syntheses reported here. In nanoparticle synthesis, the silylamide has been reported to act as a “superbase” forming a highly reactive metal intermediate (presumably M(HMDS)_*n*_), leading to accelerated nucleation rates (and correspondingly smaller nanoparticles). There are counter literature examples in which the metal halide reacted with Li(HMDS) does not form M(HMDS)_*n*_ but rather the HMDS deprotonates the oleylamine leading to a reactive metal-oleylamido complex M(RNH)_n_.^35,54^ Finally, there are also reports where HN(SiMe_3_)_3_)_2_ was used as an “additive” (small, nonstoichiometric addition), leading to changes in crystallinity, phase, and morphology of the nanosheets.^28,55,56^ For example, in the synthesis of WS_2_ from WCl_6_, the presence of the small amounts of HMDS led to a change in structure (from 1T to 2H),^57^ and the product had a similar morphology as the NdTe_3_ from Nd(HMDS)_3_. The comparison between metal silylamide (Ge(HMDS)_2_) and metal chloride (GeCl_2_) precursors in the synthesis of GeTe is informative.^58^ The metal silylamide salt exhibits rapid nucleation leading to much smaller nanoparticles, whereas the metal chloride was slower and produced nanocrystals of much larger dimension, as we see here.

In comparing the two synthetic routes reported here, we note that both syntheses include the silylamide, but the presence of chloride was unique to the thicker nanosheet synthesis. Additionally, the presence of chloride on the surface of the nanosheets is evidence that the halide coordinates to facets, and the differences in morphology reflect a change in nanocrystal growth kinetics. Chlorides have a rich literature in the synthesis of semiconductor metal chalcogenides,^59^ and the metal reagent of choice in generalized nanosheet syntheses of transition metal dichalcogenides.^60^ When used in semiconductor nanoparticle synthesis, the halide is often found in the ligand shell of the final nanomaterial^61,62^ and frequently influences the nanocrystal shape.^63,64^ The chloride has been proposed to coordinate to metal rich facets and as an X-type ligand more strongly interacts than other solution capping ligands, which are typically neutral 2-electron donors (like TOP and oleylamine). Chloride has also been used as an additive, through slow introduction via decomposition of halogenated alkanes.^65^ Chloride-assisted nanosheet (2D) growth of cubic (3D) materials has also been associated with the oriented attachment of spherical nanoparticles into nanosheets.^66^ In this mechanism, chloride can induce nanosheet thickness control through facet-specific binding and oriented attachment.^67^

Both reactions lead to nanosheets of NdTe_3_, so we believe the anisotropic bonding is clearly important to the anisotropic growth. Nonetheless, we observe a notable morphological difference between NdTe_3_ synthesized from NdCl_3_ versus Nd(HMDS)_3_, not just differences in nanosheet thickness but the nanosheets with chloride present more well-defined highly crystalline facets. The silylamide may enhance the nanocrystal nucleation for both syntheses, but the chloride appears to change the kinetics of nanosheet formation, most likely through facet-mediated growth.

### Charge Density Waves

The charge density waves in LnTe_3_ materials have been investigated using X-ray diffraction, ARPES, STEM, Raman scattering, neutron scattering, and transport measurements.^17^ In this class of materials, the charge density wave causes modulations in the pseudo-square telluride layer, which distorts to form polytelluride species along the *b* axis.^21^ This transition occurs above room temperature for lanthanides lighter than Tb and shows a *c* axis approximately 7 times larger than the unit cell. The magnitude of the distortion changes weakly for the entire series of LaTe_3_ (RE = La – Tm) and the q-vectors for the charge density wave change inversely with cell volume and range from q = 0.273c* for La to q = 0.303c* for Tm as measured by the linear distance between the nearest Bragg peaks.^68^ As temperatures are increased, these vectors increase until the charge density wave is no longer a stabilizing factor, and the telluride lattice becomes a pseudo-square again.^21^

Reports on GdTe_3_ nanomaterials have found that the charge density wave persists to the monolayer.^69^ The CDW ordering temperature (*T_CDW_*) was determined by Raman scattering from mechanically exfoliated sheets and increases (by ∼50K) as the material decreases in thickness from 155 to 10 nm.^70^ The increased *T_CDW_* is associated with an *ac* expansion (and by extension a lower q-vector) as well as a compression along the *b* axis, and interpreted as a release of chemical pressure.^66^ Consistent with this, STM studies on MBE-grown GdTe_3_ have shown a slightly decreased q-vector (associated with a higher T_CDW_), providing evidence that even to the monolayer, GdTe_3_ possesses a room temperature accessible CDW.^65^

**Figure 8:**
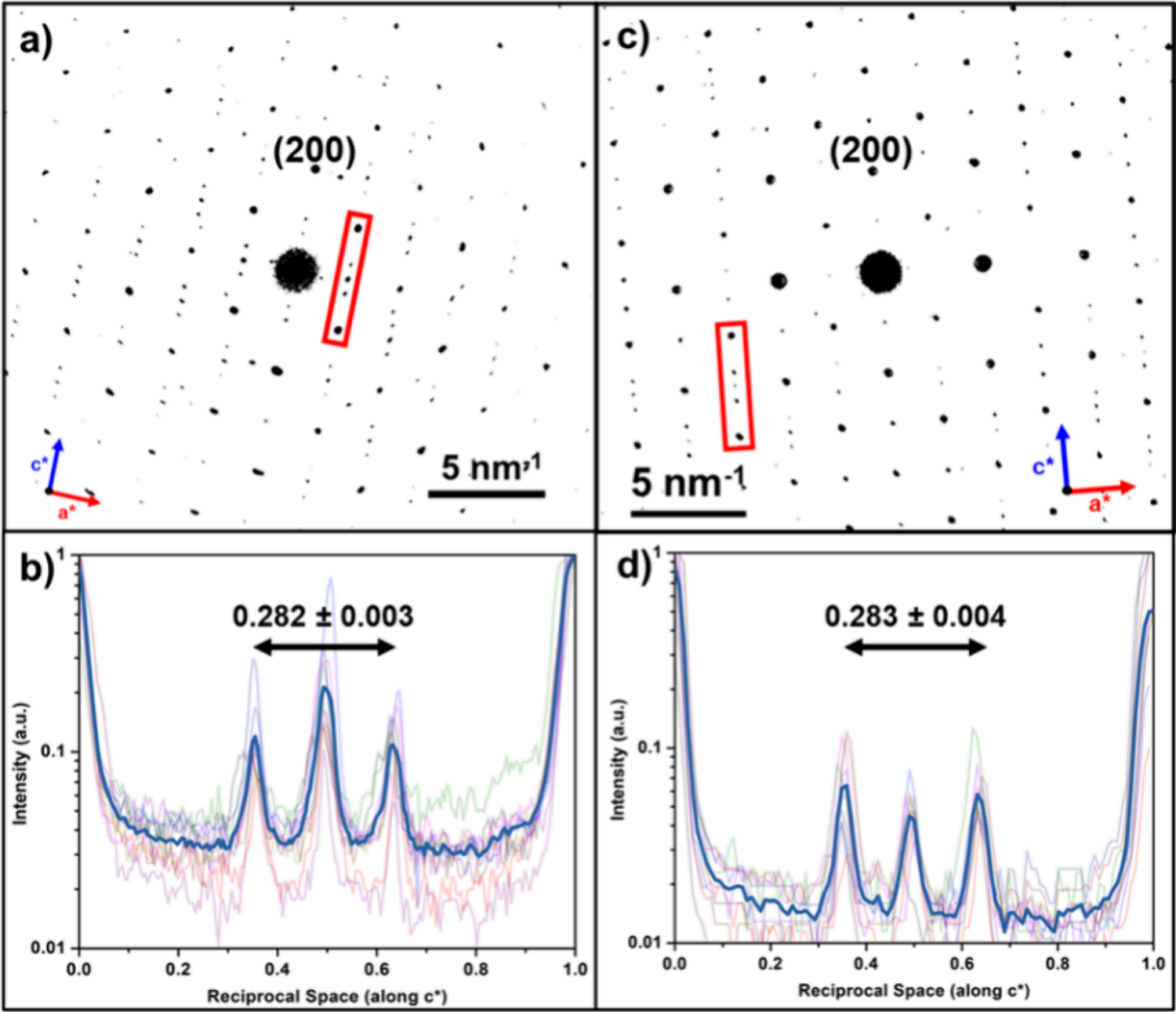
Selected area electron diffraction patterns of NdTe_3_ at room temperature synthesized from Nd(HMDS)_3_ (a) and from NdCl_3_ and Li(HMDS) (c) along the [010] zone. Intensity traces along c* for the above diffraction patterns (b, d).

The incommensurate CDW in individual NdTe_3_ nanosheets were identified by the superlattice peaks in the electron diffraction, corresponding to the expanded unit cell.^71^ Using selected area electron diffraction of both thick and thin NdTe_3_ individual nanosheets, shown in Figure 8, we found evidence of the charge density waves. The obtained diffraction patterns were processed (see SI-5 for a detailed discussion and raw images) and the q-vectors were measured and averaged. The c* direction was assigned assuming that the CDW propagates only along this direction.^21^ The literature value of q = 0.2827(3)c* was reported for room temperature bulk NdTe_3_,^21^ while we find q = 0.282(3)c* and q = 0.283(4)c*, for the thin and thick nanosheets, respectively. Due to the high T_CDW_ for NdTe_3_ (470 K),^22^ the magnitude of q vector between 100 to 400 K only changes by +0.002c*, and thus within the error of our experimental technique. Therefore, the q vector determined for our nanosheets do not notably differ from the bulk at room temperature. Despite this, the observation of characteristic superlattice peaks from colloidally grown nanosheets is strong evidence that the charge density wave is stable down to few-layer nanosheets and is reasonably insensitive to surface chemistry.

To estimate the ordering temperature of the charge density wave, we turned to variable temperature Raman experiments. In the LnTe_3_ family, the charge density wave amplitude mode involves in-plane vibrations in the [Te_*n*_] layers and varies strongly with temperature, going to zero as the critical temperature *T*_*CDW*_ is approached from below.^72^ Absent coupling with neighboring modes, the temperature dependence of this mode can be described by the Ginzburg–Landau model for a second-order phase transition
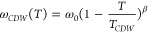
where ω_0_ is the frequency of the CDW amplitude mode at *T* = 0 K and β is a critical exponent. The CDW amplitude mode can couple to nearby phonon modes, leading to avoided crossings. This coupling can be described by a 2-level Hamiltonian with coupling constant δ
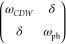
where ω_ph_ is the frequency of the temperature-independent phonon mode in absence of interactions.

**Figure 9:**
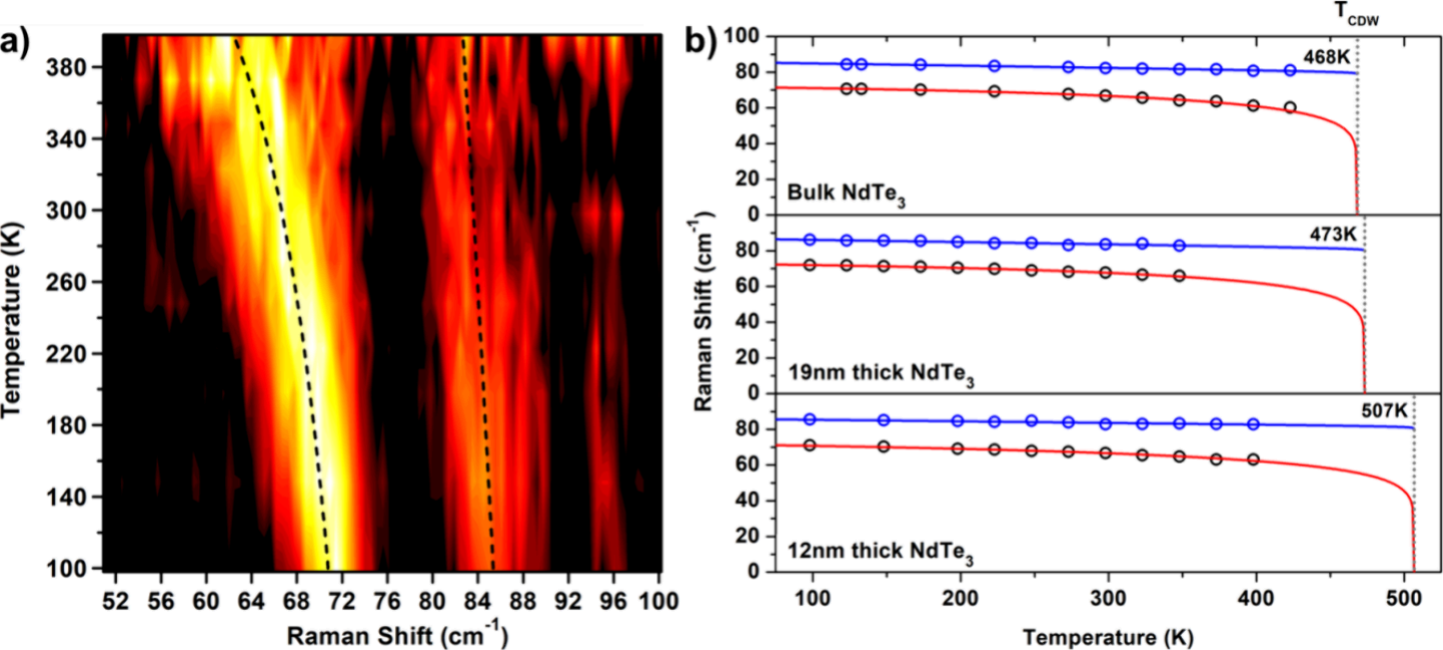
Variable temperature Raman data on bulk and NdTe_3_ nanosheets. Low-frequency Raman shift in 12 nm-thick NdTe_3_ highlighting the CDW amplitude mode around 70 cm^–1^ and a neighboring phonon mode around 85 cm^–1^ renormalized by their coupling. The fits of these peaks to the coupled CDW-phonon model are overlaid in black dashed lines (a). Temperature dependence of the Raman peaks for bulk and nanosamples (symbols) with fits to the coupled model (lines). Red lines indicate fits to the CDW amplitude mode and blue lines are the fit to the nearby phonon mode. The fits assume that the scaling parameter β is the same for the bulk and nanosheet samples (b).

In order to fit the nanosheet data to this model, we used bulk NdTe_3_ crystals synthesized by the tellurium flux method (powder diffraction data included in SI-6) as a reference. The temperature-dependent Raman spectra were measured for ensembles of both syntheses in the temperature range of 98 to 398 K. The temperature range of the data was limited based on the appearance of distinctive TeO_2–*x*_ modes,^43^ indicative of radiation-induced changes, which were detected as low as 348 K in the 19 nm-thick samples (SI-7). Both the bulk crystals and ensemble nanosheet samples exhibit two major peaks between 50 to 100 cm^–1^, as seen in the variable temperature Raman shown in Figure 9a for the thin nanosheets (the same data for the thick nanosheets and bulk NdTe_3_ are in SI-8). The lower frequency mode softens as the temperature increases, while the other mode exhibits weaker temperature dependence. We identify these modes as the coupled CDW amplitude mode and a Raman active mode involving out-of-plane buckling of the [Te_*n*_] sheets.^73^

For the bulk sample, the peak centers of the two modes were fit to the model described above, which yielded a *T_CDW_* of 468 K, consistent with the value obtained from XRD.^22^ The critical exponent β has been reported to depend primarily on the material and not the sample thickness.^66,71^ Using the fitted value of the critical exponent β from the bulk crystal data, we were able to model the nanosheet data. All other parameters were left free to minimize the root-mean-square deviation between the fit and the data. The values obtained for the parameters are provided in SI-9, along with a discussion of the uncertainties. The fits to the temperature-dependent Raman data are shown in Figure 9b, where the extracted *T*_*CDW*_ values are indicated. Under the assumption that β is fixed at the bulk value, we find an ordering temperature of 507 K for the 12 nm-thick NdTe_3_ nanosheets and 473 K for the 19 nm-thick nanosheets. This variation in *T*_*CDW*_ with thickness is consistent with reports on individual GdTe_3_ nanosheets that indicate that the ordering temperature remains relatively constant for sample thicknesses down to about 20 nm and then increases below 20 nm.^66^ The presence of the charge density wave also suggests that any oxidation of the materials is surface limited.

### Nanosheet Magnetism

In the bulk, NdTe_3_ is an antiferromagnet with a Néel temperature of 3.2K and moment of 3.6 μ_B_ per Nd. Like other tritellurides, NdTe_3_ exhibits high magnetic anisotropy due to antiferromagnetically coupled moments within the *ac* plane, and only a small magnetic component along the unique *b* axis.^24^ Due to the high structural similarities between the lanthanide polytellurides (LnTe_*x*_ where *x* = 2, 2.5, 3), comparisons of the magnetic orientation, strength, and moment have been fruitful in determining how the Te layer influences the magnetism in LnTe_3_. The magnetic character in the LnTe_3_ materials is thought to be influenced by the presence of RKKY interactions between the [LnTe]^+^ block and metallic [Te_*n*_]^−0.5^ layers.^24^ The lanthanide tritelluride family of materials are fascinating to study on the nanoscale due to the apparent sensitivity of the magnetic environment to electronic changes within the tellurium layer.

One of the pressing questions in 2D magnetism is to determine trends in thickness-dependent magnetic ordering temperature or orientation, and guidelines for what nanosheet thickness is the threshold below which deviations from the bulk can be expected. A review of the literature provides many counter examples. For example, the Néel temperature in exfoliated FePS_3_ is relatively constant from bulk down to the monolayer.^74^ By contrast, a small but steady decrease in *T*_c_ in Fe_3_GeTe_2_ is observed until 4 nm (5 ML), where a sudden drop in *T*_c_ and crossover from 3D to 2D Ising ferromagnetism was found.^75^ Room-temperature ferromagnetism in CrTe_2_ is found down to 10 nm (17 ML), below which *T*_c_ decreases,^7^ whereas Cr_2_Te_3_ exhibits an increase in *T*_c_ to room temperature for nanosheets of below 10 nm^76^ While colloidally synthesized nanosheets currently cannot be used to explore those systems that only exhibit novel magnetism as a monolayer, such as the room temperature ferromagnet VSe_2_,^77^ many of the magnetic materials reported exhibit measurable changes in the nanoscale. Thus, colloidal synthesis of magnetic nanosheets may provide significant contributions to our understanding of 2-dimensional magnetism.

**Figure 10:**
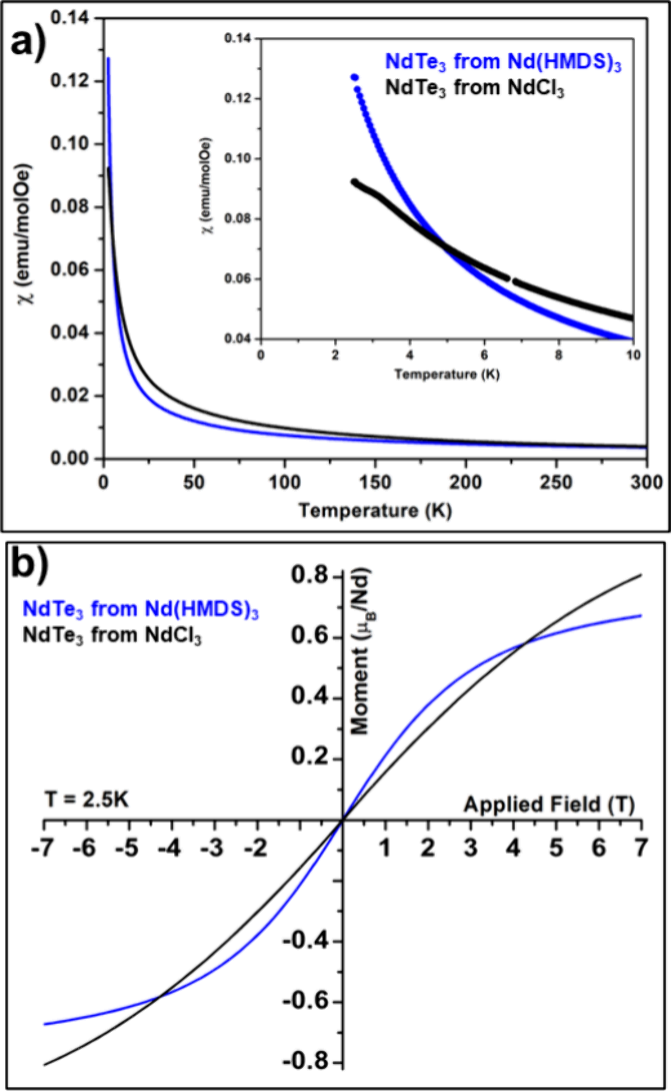
Magnetic characterization of NdTe_3_ nanosheets. Field-cooled susceptibility vs temperature at 0.1 T (a) and moment vs field plots for NdTe_3_ from Nd(HMDS)_3_ (thin, blue) and from NdCl_3_ (thick, black) recorded at 2.5K (b).

Taking advantage of the high yields from colloidal synthesis (5–10 mg), we were able to measure the magnetic properties of the two types of nanosheets using superconducting quantum interference device (SQUID) magnetometry. While the average population of nanosheet thickness for the two materials is relatively close (12 and 19 nm), we found distinct differences in the magnetic susceptibility of powders from multiple syntheses. Measurements were made on samples produced by centrifugation and collection of the precipitate. The reported χ(*T*) data for single crystals exhibit a clear peak in the χ vs *T* at 3.2 K for parallel to the *ac* plane but not in the perpendicular orientation. The thick nanosheet exhibited a χ(*T*) with a Néel temperature where the small peak in the dχ/d*t* suggested a value of 3.3–3.8 K, close to the bulk value; however, there was no such peak for the thin NdTe_3_, as compared in the inset of Figure 10. There was far less preferred orientation observed by PXRD in the thin NdTe_3_; however, the χ(T) for the thin nanosheet had the appearance of a paramagnet, and derivative of the spectra show no evidence of ordering (the derivative spectra provided in SI-10). In the lanthanide polytelluride family, as the tellurium becomes more anionic, the magnetic coupling weakens, as a result of increased ionicity and the opening of a bandgap.^24^ For example, the related reduced polytelluride, NdTe_1.89_, is a paramagnetic semiconductor with a theta of −4.9K.^78^ This effect might have been expected for the thick NdTe_3_ with the terminating layer [Nd^3+^Te^2–^]^+^[Cl]^−^, which behaved closer to the bulk but may explain the weaker peak at the Néel temperature.

We used a temperature range above 200 K for the Curie–Weiss analysis, in order to compare to the bulk literature values, and the recommended temperature range for lanthanides.^79^ Based on the Curie–Weiss fit of the high temperature range, both nanosheets had a calculated μ_eff_ within the range expected for Nd^3+^ as provided in Table 2. Crystal field effects can lead to a slightly lower moment,^80^ but both thick and thin NdTe_3_ nanosheets had the same calculated moment within experimental error. The Weiss constant (θ_CW_) for lanthanides can contain contributions from populated low-lying excited states leading to an overestimated strength of the interactions.^79^ Compared to the bulk value of θ_CW_ of −20 K (the average from measurements parallel and perpendicular to the *b* axis in single crystals), the calculated θ_CW_ for 19 nm-thick NdTe_3_ was −38 K (±4 K). The calculated θ_CW_ was −54 (±3) K for the 12 nm-thick NdTe_3_ (see Figure SI-11 for Curie–Weiss fit). An increase in the magnitude of the theta value is often associated with a higher transition temperature, but the absence of magnetic ordering in the thin NdTe_3_ could imply that the material is becoming magnetically frustrated, as has been seen in other antiferromagnetic nanosheets.^81^ What is notable is that exposure to air over a period of 24 h of the same sample, the theta becomes increasingly negative. This cannot be explained by an impurity in the synthesis, and we suggest that this is may be attributed to the reduced thickness of unoxidized NdTe_3_. This effect has been observed in the increase in *T_CDW_* with aging as observed for RTe_3_.^47^

**Table 2:** Magnetic Data Summary

**material**	**moment (μ**_**B**_**)**	**θ**_***CW***_**(K)**	***T***_**N**_**(K)**
bulk NDTe_3_			
parallel to *ac* plane	3.59	–13.6	
parallel to *b*	3.69	–27.1	3.2
19 nm-thick NdTe_3_	3.26	–38 ± 2	∼3
12 nm-thick NDTe_3_	3.64	–55 ± 5	N/A

The M vs H data for bulk NdTe_3_ indicates that the material does not saturate and there is a metamagnetic transition around 5 T.^24^ We found that the magnetization vs field measurements of the two materials differ: thick NdTe_3_ nanosheets show a near linear moment as a function of applied field (typical of an antiferromagnet), while the thin NdTe_3_ thin nanosheets exhibit a soft ‘s’ curve. While this shape could indicate ferromagnetic coupling, there is no evidence of hysteresis or an increased saturation magnetization making this conclusion unlikely. Therefore, we attribute the magnetic change to a transition from bulk antiferromagnetism to paramagnetism with Brillouin behavior on the nanoscale.^82^ The changes observed here are unlikely to be due to the type of symmetry-related changes that can occur near to the monolayer, which would be 1.2 nm for NdTe_3_.

## Conclusions

The solution synthesis of the lanthanide tritelluride, NdTe_3_, formed crystalline nanosheets in high yields. The differences between the syntheses developed from Nd(HMDS)_3_ compared to the material from NdCl_3_ suggest that the halide plays an important role in the kinetics of nanocrystal growth, leading to differing morphologies. Although we did not develop the synthesis further for size control over a wider range of nanosheet thicknesses, the syntheses did allow for a comparison of two different thicknesses of nanosheets. Populations of both types of nanosheets show charge density wave ordering with temperatures comparable to or higher than the bulk. The fact that the magnetic properties differ between the thick (19 nm) and thin (12 nm) nanosheets suggest a marked transition in the magnetism within a narrow range in thickness. The synthesis is reproducible and high yielding enough that we plan to use these nanosheets to investigate the magnetic ordering by neutron diffraction methods.

## Methods

### Materials

Nd((Me_3_Si)_2_N)_3_ (98%, Thermo), NdCl_3_ (99.9%, alfa), lithium bis(trimethylsilyl)amide (97%, Sigma), neodymium (>99%, Sigma), tellurium (99.8%, sigma), trioctylphosphine (97%, Sigma), diphenylphosphine (98%, Sigma), anhydrous dichloromethane (≥99.8%, Sigma), anhydrous hexanes (≥99%, Sigma), and anhydrous toluene (99.8%, sigma) were used as received. Oleylamine (OLA) (>70%, Sigma) was distilled under vacuum to a clear and colorless liquid then stored in the glovebox.

### TOP-Te Synthesis

In a nitrogen glovebox, tellurium metal (2.54g, 20 mmol) and 97% trioctylphosphine (20 mL, 44.8 mmol) were added to a Schlenk flask. This was heated and stirred under argon for 2.5 h at 240 °C. The resulting yellow liquid was stored in the glovebox.

### Synthesis of 12 nm NdTe_3_ Nanosheets

In a nitrogen glovebox, Nd((Me_3_Si)_2_N)_3_ (0.044g, 0.07 mmol), 1 M TOP-Te (1 mL, 1 mmol), TOP (1 mL), Ph_2_PH (1 mL), and OLA (2 mL) were added in order to a flask then fitted with a condenser and gas adapter. On a Schlenk line, the solution was heated at 100 °C under vacuum for 20 min and then was heated under argon to reflux (∼360 °C) for 1 h. This formed a dark gold/black colloidal solution with a golden film coating the interior of the flask. Upon cooling, the solution was dispersed in 3 mL of hexanes and precipitated in 40 mL of dichloromethane in an inert atmosphere. This was centrifuged at 4500 rpm (471 rad/s) for 5 min and the solid pellet was collected. The product was washed again with 3 mL of hexanes and 40 mL of dichloromethane. This was centrifuged again and the solid was collected and matched the PXRD reference pattern for NdTe_3_ (ICSD170558).

### Synthesis of 19 nm NdTe_3_ Nanosheets

In a nitrogen glovebox, NdCl_3_ (0.018g, 0.07 mmol), Li(Me_3_Si)_2_N (0.050g, 0.3 mmol), 1 M TOP-Te (1 mL, 1 mmol), TOP (1 mL), Ph_2_PH (1 mL), and OLA (2 mL) were added in order to a flask while stirring with a glass coated stir bar and then fitted with a condenser and gas adapter. The solution was heated at 100 °C under vacuum with stirring for 20 min, then stirring was stopped, and the reaction was heated under argon to reflux (∼360 °C) for 1 h. This formed a dark gold/gray colloidal solution with a golden film coating the interior of the flask. Upon cooling, the solution was dispersed in 3 mL of hexanes and precipitated in 40 mL of dichloromethane in an inert atmosphere. This was centrifuged at 4500 rpm (471 rad/s) for 5 min and the solid pellet was collected. The product was washed again with 3 mL of hexanes and 40 mL of dichloromethane. This was centrifuged again and the solid was collected and matched the PXRD reference pattern for NdTe_3_ (ICSD170558).

### Synthesis of Bulk NdTe_3_

NdTe_3_ single crystals were synthesized according to the literature procedure with minor modification.^20^ 108 mg of Nd and 1.18g of Te were added to a graphitized quartz tube and then topped with a plug of quartz wool. The tube was sealed and heated vertically to 900 °C over 4 h with the metals below the quartz plug. The furnace was kept at this temperature for 18 h and then cooled to 550 °C at 2 °C/h. Upon reaching 550 °C, the tube was flipped and centrifuged. Under an inert atmosphere, gold crystals were collected and purified of residual Te by heating at 410 °C for >3 h in evacuated quartz. The ∼100 mg of golden crystals were referenced to the literature PXRD pattern for NdTe_3_ (ICSD170558) as shown in SI-6.

### Characterization

X-ray powder diffraction patterns were obtained using a Rigaku Ultima IV X-ray powder diffractometer with Cu Kα radiation at 40 kV and 30 mA and a D/teX silicon strip detector. Rietveld refinements were performed using the Generalized Structure and Analysis System (GSAS) and refined on unit cell, particle size, and thermal parameters. Samples were prepared for SEM and TEM, measurements by drop casting dilute nanomaterial solutions in hexanes or toluene on carbon-coated copper TEM grids, single crystal Si wafers for AFM, and glass slides for Raman. High-resolution TEM (HRTEM) and energy-dispersive X-ray spectroscopy (EDS) analyses were performed on a JEOL JEM-2100F FEG TEM instrument operated at 200 kV at the Advanced Imaging and Microscopy Lab at the University of Maryland. Scanning electron microscopy (SEM) images were taken with a Zeiss SUPRA 55-VP scanning electron microscope, at an acceleration voltage of 20 kV with an in-lens detector.

AFM and Raman samples were prepared by drop casting a dilute solution of nanoparticles dissolved in hexanes or toluene onto a (100) silicon wafer. Raman spectroscopy was performed with a Horiba Raman microscope equipped with a 532 nm laser at 10 μW power and a 1800 line/mm grating and calibrated against a diamond standard. The instrument was interfaced with an Olympus BH2-UMA optical microscope, and a magnification factor of 50× was used. Spectra were recorded in extended scan mode from 50 to 200 cm^–1^ and analyzed using the WiRE 2.0 software package. Variable temperature Raman measurements were performed under a nitrogen atmosphere using a Linkam heating/cooling stage. The surface topography was acquired with an NTEGRA scanning probe microscope (NT-MDT) operated in semicontact/tapping mode. The probe is made from single-crystal silicon with a nominal cantilever spring constant of ∼12 N/m.

Magnetic susceptibility was obtained on a Quantum Design MPMS3 SQUID magnetometer. Data were collected using a temperature sweeping mode from 2.5 to 300 K at 1000 Oe (0.1 T) under both zero-field cooled (ZFC) and field-cooled warming (FCW) conditions. The Curie–Weiss analysis was done on FC data at 1000 Oe (0.1 T) between 200 and 300 K. Magnetic hysteresis data was collected at 2.5 K from −7 to 7 T. All data were corrected for diamagnetic contributions using Pascal’s constants.^83^ FTIR measurements were recorded in the range of 400–4000 cm^–1^, from pressed pellets in KBr on a PerkinElmer Spectrum Two FTIR instrument.

X-ray photoelectron spectroscopy (XPS) was performed using a Kratos Axis Ultra system with a monochromatic Al Kα excitation source operating at 15 kV and 10 mA. NdTe_3_ samples were drop cast on a Si wafer and pumped to a base pressure of <2.0 × 10^–9^ Torr (2.7 × 10^–7^ Pa) before measurement. The detector was at a photoelectron takeoff angle of 90° to the surface and a pass energy of 20 eV was used. Samples were sputtered with an Ar^+^ ion gun for 5 min prior to measurement. Binding energies were normalized to C 1s at 284.6 eV. Data analysis was conducted with Shirley backgrounds using Voigt functions in CasaXPS.

## Supporting Information Available

Reaction intermediates characterized by ^31^P NMR can be found in SI-1; XPS identifying Te on unsputtered materials in SI-2; additional TEM of NdTe_3_ from the two reactions in SI 3 and 4, with electron diffraction in SI-5; bulk NdTe_3_ characterization is in SI-6, with Raman data in SI-8; Raman data of heated 19 nm-thick NdTe_3_ is in SI-7; for the CDW studies, the fitting parameters in SI-9 and magnetic characterization can be found in SI-10 and 11 (PDF)

## ABBREVIATIONS

PXRD: powder X-ray diffraction
TEM: transmission electron microscopy
AFM: atomic force microscopy
SEM: scanning electron microscopy
EDS: energy-dispersive X-ray spectroscopy
XPS: X-ray photoelectron spectroscopy
