# Supplementary material for: Solution Synthesis of NdTe3 Magnetic Nanosheets
Source: Chem Mater. 2024 Jul 4;36(14):7056–68. doi: 10.1021/acs.chemmater.4c01362 (PMC11270740; doi:10.1021/acs.chemmater.4c01362)
Supplement: Supplementary file 1 — cm4c01362_si_001.pdf [file cm4c01362_si_001.pdf]

Supplemental Information for:

Solution Synthesis of $NdTe_3$ Magnetic Nanosheets

Joel Swanson, [1] *Salah Eddin El Jamal,* [1] *Tyler Hartman,* [1] *Orlando C. Stewart,* [1] *Priscilla Glaser,* [1] *Adam J. Biacchi,* [2] *DaVonne Henry* [3] *Amy Liu,* [3] *Sarah L. Stoll*[1]*

[1]Department of Chemistry, Georgetown University, 37th and O Sts. NW, Washington DC 20057 United States.

[2]Nanoscale Device Characterization Division, National Institute of Standards and Technology (NIST), 100 Bureau Dr., Gaithersburg MD 20899, United States

[3]Department of Physics, Georgetown University, 37th and O Sts. NW, Washington DC 20057 United States.

Table of Contents

SI-1 ^{31}P NMR studies of Reaction (1) using $La(HMDS)_3$ 2

SI-2 Tellurium 3d pre-sputter XPS for 19nm thick $NdTe_3$ (a) and 12nm Thick $NdTe_3$ (b) 2

SI-3 TEM survey of $NdTe_3$ from $NdCl_3$ and Li(HMDS) 3

SI-4 TEM Survey of $NdTe_3$ from $Nd(HMDS)_3$ 3

SI-5 Unprocessed $NdTe_3$ nanosheet Electron Diffraction patterns 4

SI-6 Bulk $NdTe_3$ PXRD Pattern 4

SI-7 Raman of 19nm Thick $NdTe_3$ collected at 398K 5

SI-8 Bulk(left) and 19nm thick (right) $NdTe_3$ Variable Temperature Raman Data and Fitting. 5

SI-9. Fitting Parameters for Model of Coupled CDW Amplitude Mode and Phonon Mode 6

SI-10 Derivative of χ vs T for 19nm thick $NdTe_3$ (a) and 12nm thick $NdTe_3$ (b) 7

SI-11 Curie Weiss Analysis of $NdTe_3$ Nanosheets 7

References 7

SI-1 ^{31}P NMR studies of Reaction (1) using $La(HMDS)_3$

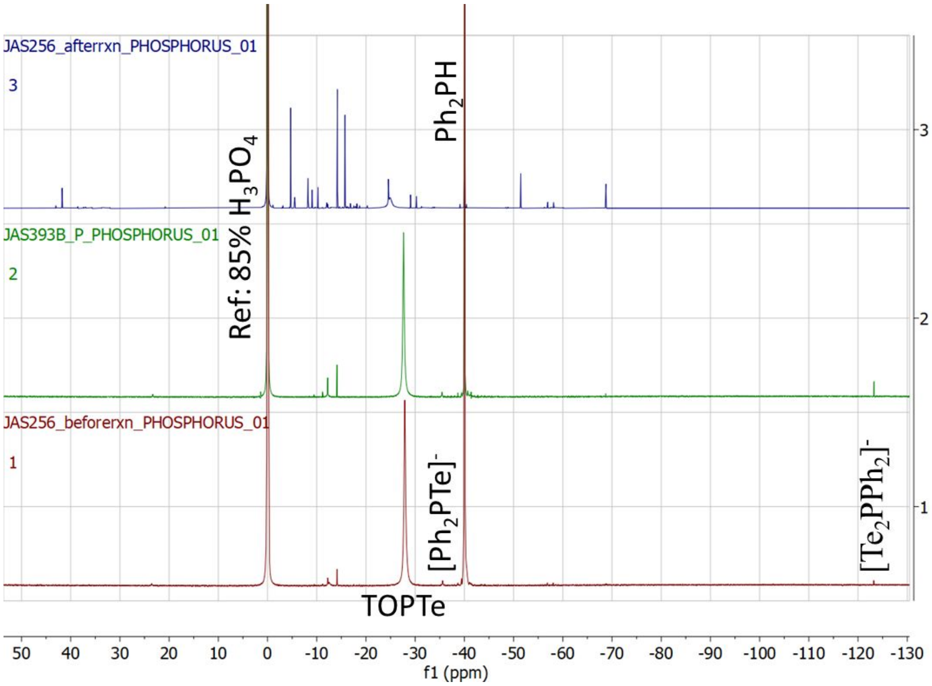

$La(HMDS)_3$ was used as a synthetic analogue to Nd^{+3} for NMR studies of the reaction solution. The bottom NMR is before the reaction starts, middle is after heating at 100°C for 30 min and top is after reaction. Characteristic tellurophosphinate peaks[1] of $[Ph_2PTe]^-$ (-35.6) and $[Ph_2PPTe_2]^-$ (-123.2) appear and grow until the reaction is complete suggesting that these are some active species in the solution. The peak of $Ph_2P\text{-}PPh_2$ at -14ppm[2] continuously grows through the reaction.

SI-2 Tellurium 3d pre-sputter XPS for 19nm thick $NdTe_3$ (a) and 12nm Thick $NdTe_3$ (b)

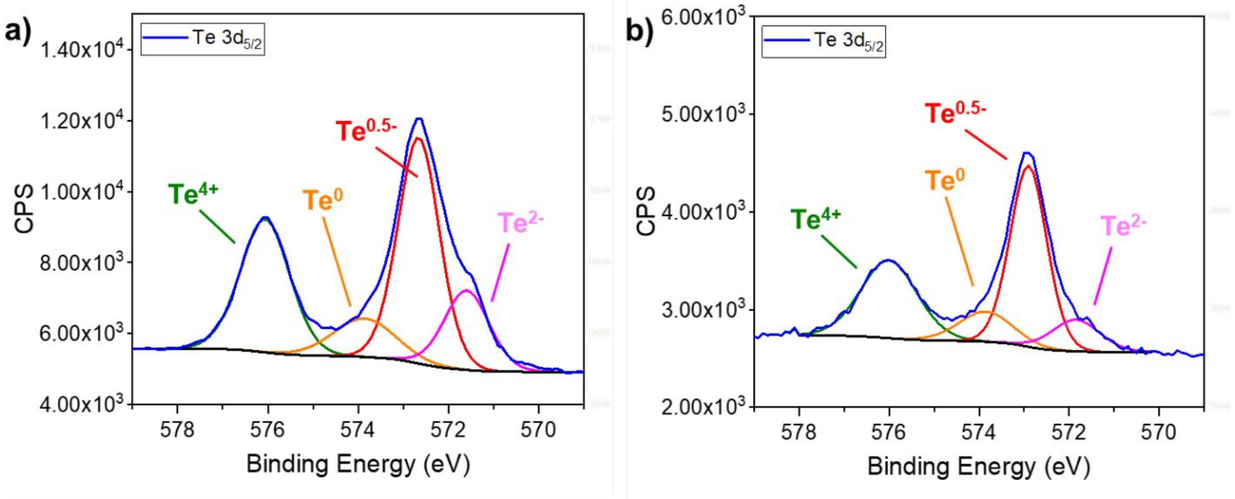

SI-3 TEM survey of $NdTe_3$ from $NdCl_3$ and Li(HMDS)

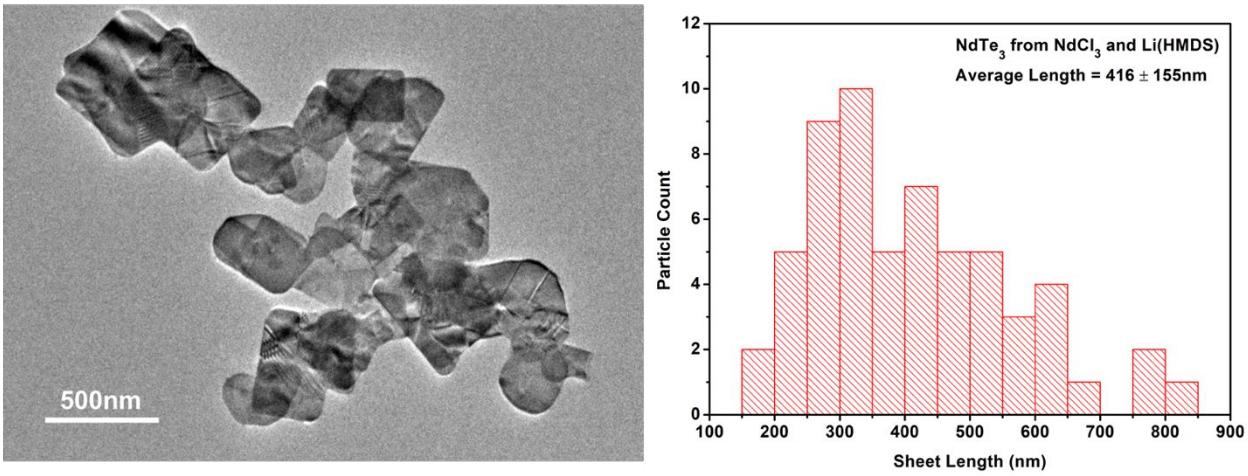

SI-4 TEM Survey of $NdTe_3$ from $Nd(HMDS)_3$

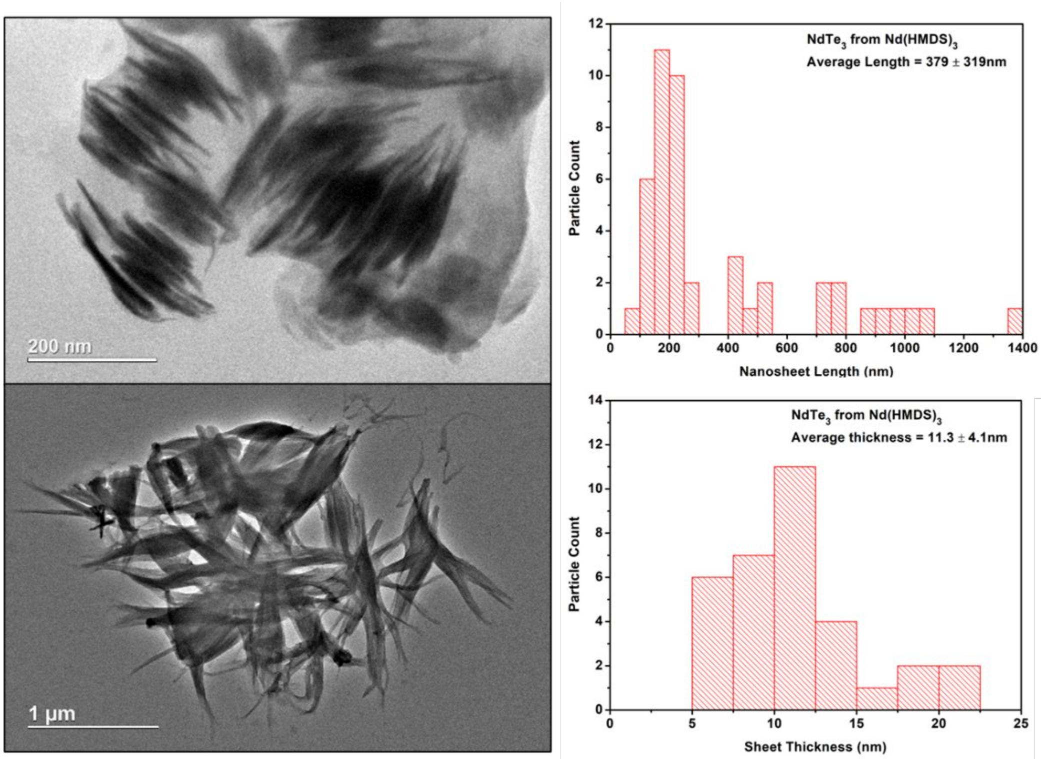

SI-5 Unprocessed $NdTe_3$ nanosheet Electron Diffraction patterns

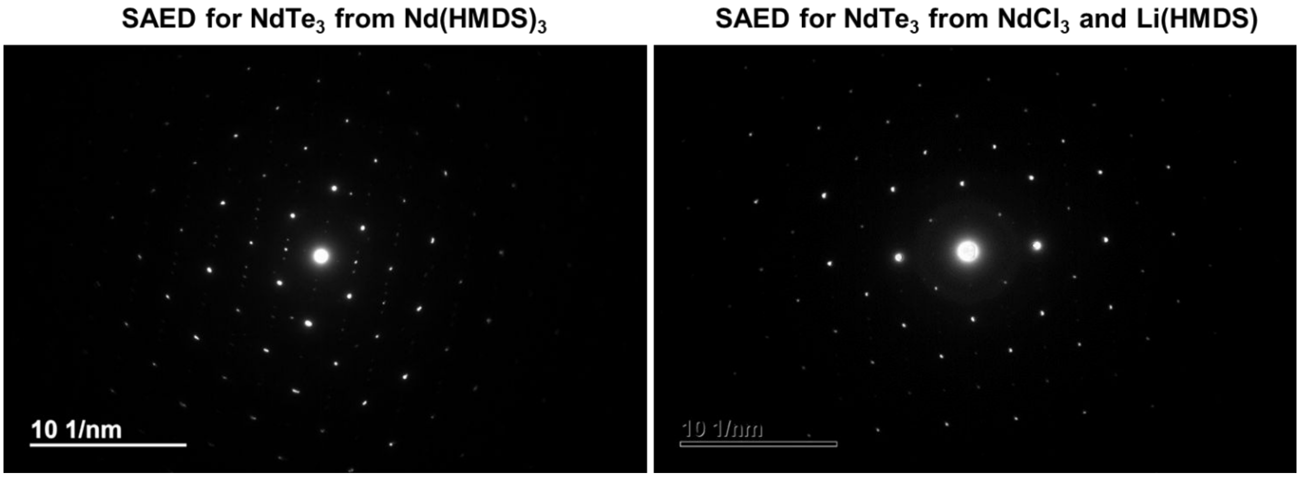

Selected area electron diffraction images were processed with a local threshold Phansalkar method to highlight the superlattice peaks, the intensity of these images was then reversed to make figure 8a and 8c. Superlattice traces were performed on the unprocessed image and q-vectors were calculated for each trace before averaging (n = 10).

SI-6 Bulk $NdTe_3$ PXRD Pattern

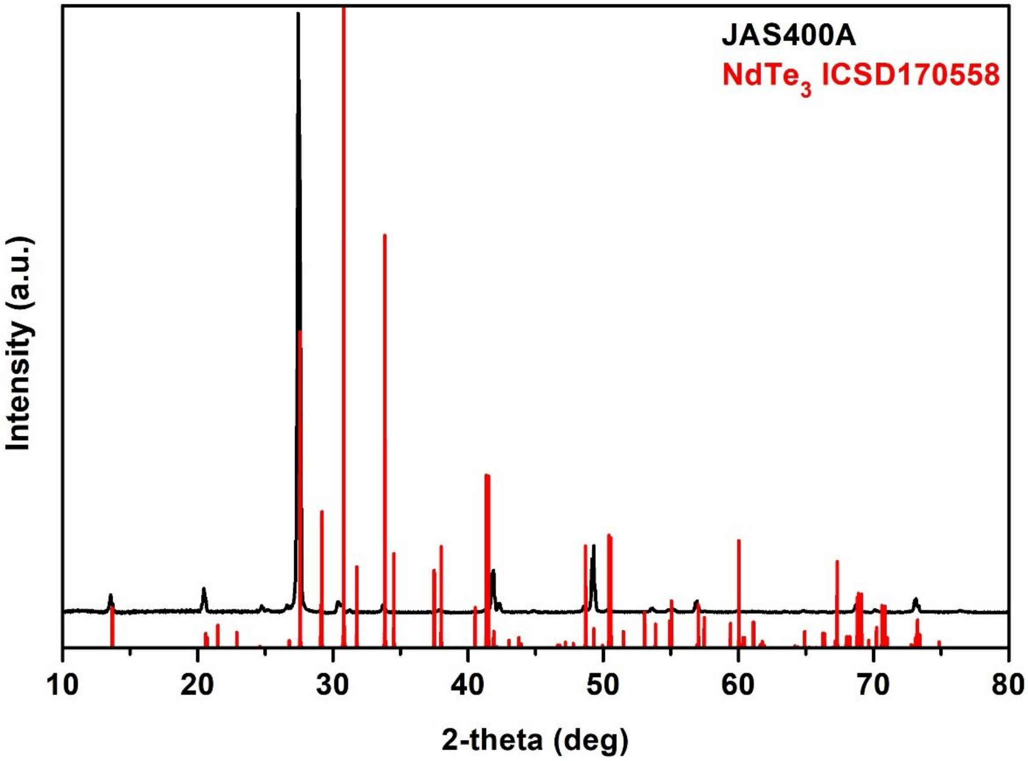

SI-7 Raman of 19nm Thick $NdTe_3$ collected at 398K. This peak has been ascribed to TeO_2 and Te in the literature.[3]

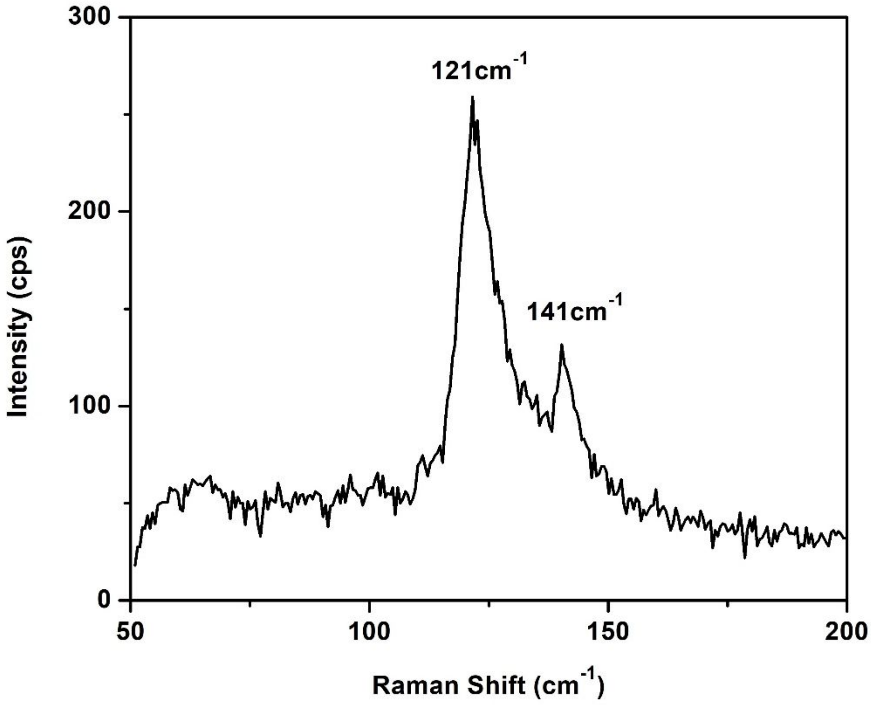

SI-8 Bulk(left) and 19nm thick (right) $NdTe_3$ Variable Temperature Raman Data and Fitting.

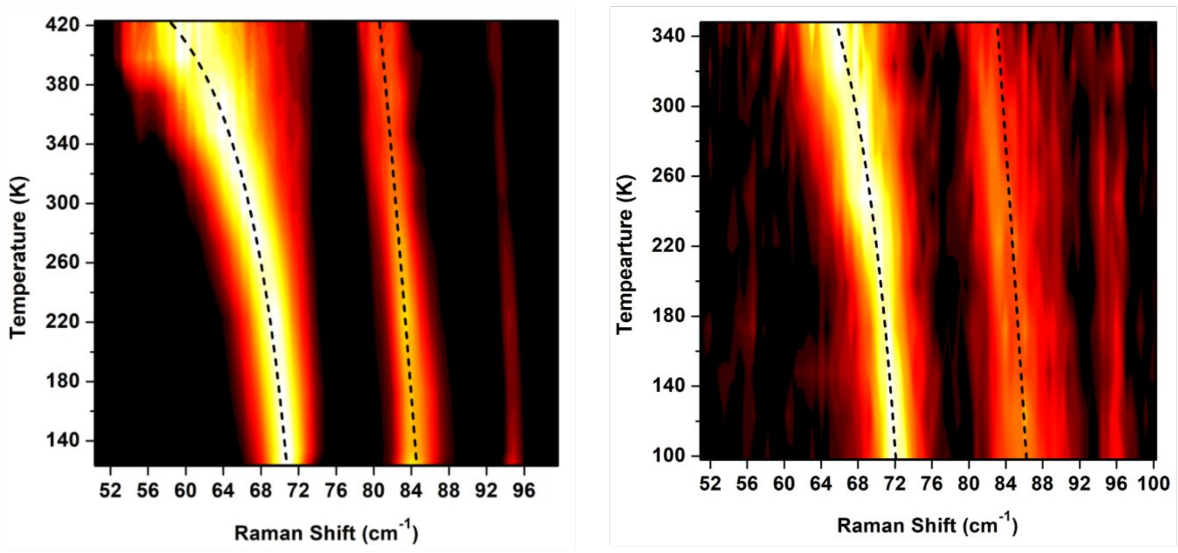

SI-9. Fitting Parameters for Model of Coupled CDW Amplitude Mode and Phonon Mode

The parameters from fitting the coupled model described in the Charge Density Wave section to three sample thicknesses are given in the table below. Confidence ranges for each parameter were calculated by holding all others fixed at their optimized values and finding the range over which the RMS error changes by less than 10%.

Parameter	Bulk $NdTe_3$	19 nm Thick $NdTe_3$	12 nm Thick $NdTe_3$
T_{CDW} (K)	468<br>(457-483)	473<br>(447-507)	507<br>(487-531)
ω_0 (cm^{-1})	80.0<br>(79.0-81.0)	81.0<br>(80.2-81.8)	78.4<br>(77.7-79.1)
β	0.119<br>(0.109-0.128)	0.119<br>(0.106-0.132)	0.119<br>(0.109-0.129)
ω_{ph} (cm^{-1})	78.3<br>(77.4-79.1)	79.4<br>(78.6-80.1)	79.8<br>(79.1-80.4)
δ (cm^{-1})	6.91<br>(6.36-7.45)	7.06<br>(6.63-7.48)	7.11<br>(6.68-7.54)
**RMSE**	0.16	0.30	0.34

The above table assumes that the critical exponent β is independent of sample thickness. To investigate the sensitivity of T_{CDW} to this assumption, we fix β to the extremes of the confidence range for each thickness and allow other parameters to vary. The resulting ranges of T_{CDW} for the nanosheet samples are shown below.

	T_{CDW} (β_{low})	T_{CDW} (β_{high})
**19 nm**	451 K	496 K
**12 nm**	492 K	523 K

SI-10 Derivative of χ vs T for 19nm thick $NdTe_3$ (a) and 12nm thick $NdTe_3$ (b)

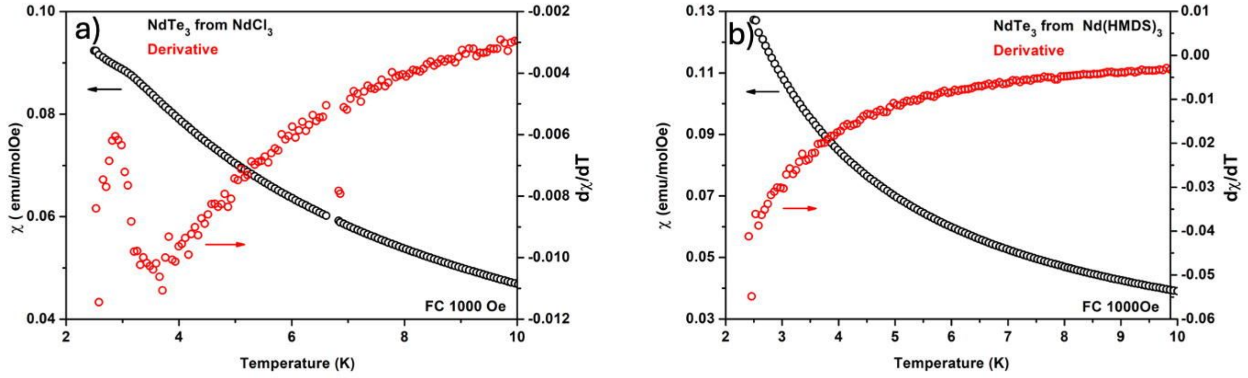

SI-11 Curie Weiss Analysis of $NdTe_3$ Nanosheets

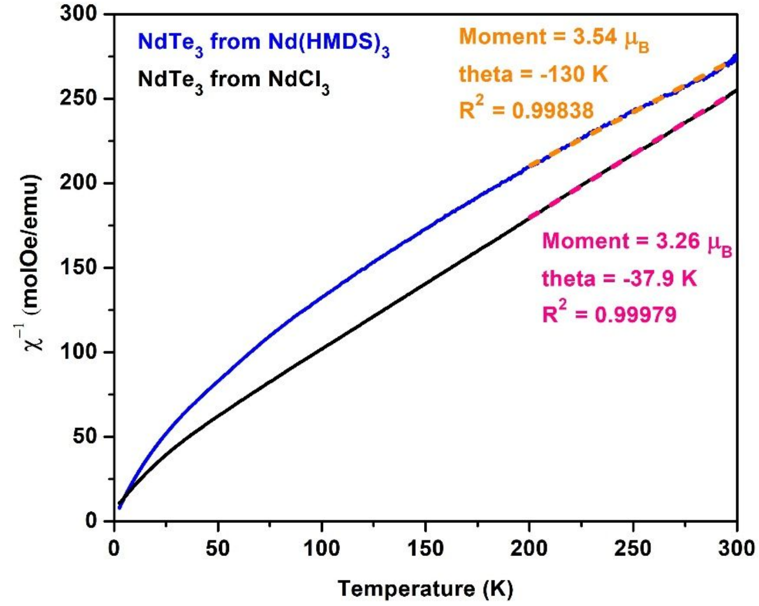

References

[1] Davies, R. P.; Martinelli, M. G.; Wheatley, A. E. H.; White, A. J. P.; Williams, D. J. Structural Studies of Lithium Telluro-and Seleno-Phosphorus Compounds. *Eur J Inorg Chem* **2003**, *2003* (18), 3409–3416.

[2] You, Q.; Zhang, J.; Zhang, F.; Cai, J.; Zhou, X. Cooperative Rare-Earth/Lithium-Mediated Conversion of White Phosphorus. *Chemistry–A European Journal* **2023**, *29* (22), e202203679.

[3] Manjón, F. J.; Gallego-Parra, S.; Rodríguez-Hernández, P.; Muñoz, A.; Drasar, C.; Muñoz-Sanjosé, V.; Oeckler, O. Anomalous Raman Modes in Tellurides. *J. Mater. Chem. C* **2021**, *9* (19), 6277–6289.
